# Judging the quality of (fake) news on the internet

**DOI:** 10.1007/s11299-020-00249-x

**Published:** 2020-08-09

**Authors:** Stefan Rass

**Affiliations:** Institute for Applied Informatics, Universitätsstrasse 65-67, 9020 Klagenfurt, Austria

**Keywords:** Fake news, Influence, Social media, Artificial intelligence, COVID-19

## Abstract

The only reliable remedy against anxiety is information, and reliable information and news are of crucial value in times of crises, such as COVID-19. Contemporary social media offers almost everyone a platform to publish one’s own thoughts, opinions, political statements and others, some of which may gain significant interest of others and thereby become so called “influencers”. This role has in the past been held by news agencies primarily, but this role is increasingly adopted also by private people and among them, also some who do not necessarily adhere the high standards of good journalism or scientific ethics. These give rise to fake news, spreading as unconfirmed rumors and possibly causing dramatic impacts to a society. With information available almost everywhere in the internet today, the distinction between good and bad sources has become a challenge, and highly difficult task. Even more intricate is the question of verifying information against multiple independent sources. If many people say something, does this make it true or any more plausible? Do we need to trust information in lack of better information? Is it possible to judge information and make our own opinion about its validity, quality, relevance or usefulness for our own business? This article shall provide pointers towards answers to the above questions. We discuss some technical means of judging the quality of information and what anyone, even without much technical background can do to avoid falling victim to fake information and fake news.

## Introduction

Ever since the scandals around Cambridge Analytica, it has been recognized that the power of social media is useable to control people’s opinions by systematic spread of particularly crafted information to make people believe what they should believe. Various concepts like the “filter bubble” or others have been scientifically studied to describe the phenomenon that people may not be confronted with the full range of information, but only with a carefully selected subset of news that suits their interests, and possibly also their opinions. While there is nothing bad per se about giving people information that they are interested in, the ethical line not to be crossed is the point where the information that is given omits important information with the aim of “controlling” people’s opinions, fears, wishes or others. This would be fiddling with people’s free will, and leaving the philosophical questions aside here, let us adopt a technical perspective about how naturally grown internet connectivity has given birth to “Influencers”, and what theory and technology related to artificial intelligence can do here.

First, let us distinguish a few terms for clarity (Rubin et al. 2015): false information is not necessarily the same as fake news, but the difference may be subtle: there is *unverified information*, which merely means that the information is passed onwards without verifying its quality or truth. There is not necessarily a manipulative intention behind this; in many cases, it is about gaining visibility (“clicks”, “likes”, …) or for economic purposes (say, to make ads visible more widely). Then, there is *satire* (or also *sarcasm*), which is also wrong information, but formulated in, often spoken, language that makes recognizing the incorrectness easy, so as to transport an underlying truth on a meta-level. Likewise, there is normally no manipulative intention behind. Exactly this distinguishes *fake news*, as being unverified (and in some cases even unverifiable) information, but coming in a jargon or language that makes it look trustworthy to serve a manipulative hidden agenda.

Fake news can come in a variety of forms (Tan 2018), including the following: intended forgery (e.g., hand-crafted to look most realistic, whereas it is known to be false), selection of facts (e.g., taken out of the context or with details missing to change the understanding into wrong impression), conspiracy theories (e.g., presenting clear guilt of somebody about something, thereby avoiding all complexity of critical thinking), immoral arguments (e.g., alluding to the freeness of opinions), or manipulated audio/video. These are only a few selected forms of fake news, and not all of them are easy to discover.

In some cases, we can discover a fake news by asking for more details about it. Intentional (not social) lying is very difficult, since the wrong information has to be consistent with a potentially huge number of related facts. Truth does not suffer from this problem, but a liar has to align its claims with an unlimited number of facts that could be brought into the picture. Thus, a first recommendation to recognize fake news is to *ask for their origin and underlying evidence*. For example, if an article claims a mortality rate of x% of COVID-19, one should ask about how this number has been calculated. There are different ways in which a mortality rate could be defined, for example, do we count people having had a disease at the time of death, or only those that were really dying from the disease (and not for a coincidental other reason). So, the mere statement that “the mortality rate is x” by itself is generally insufficient. An article speaking about such information should—to avoid the “fact selection” issue mentioned above, at least give the underlying numerical data and be specific on the details of the statistics that have been done.

## Tracing the sources of information and the existence of “Influencers”

Serious news articles give clear information on the writer, whose identity is verifiable and whose background knowledge on the topic is documented somewhere. It is the authors response to assure the validity and have the underlying facts undergo a quality check, since the actual source behind is usually not disclosed (even protected in good journalism). For web pages, an imprint, date of last update and host are the minimum requirements. Absence of such information is not necessarily a sign of fake news, but an indication to be at least cautious. For images, it pays to use the “image reverse search” that many contemporary online search engines offer, to see where the picture occurred elsewhere in the web, and ultimately to see where it came from first.

Many people get their news and information from search engines like Google. But where does Google get its information from? Roughly speaking, Google ranks pages according to the lot of links leading there. Similarly, science nowadays judges research quality based on how often an article is cited. Alas, the number weblinks, “likes” (in social media) or citations is not an indicator of correctness, truth or soundness, since these numbers do not tell the context of the citation (a link can call the reference valuable, false, or even dangerous to believe, but irrespectively of this would count as a +1 into the citations). The study of citation graphs and the internet topology uses random graph theory, and has made interesting discoveries: for example, some citation graphs exhibit small-world phenomena (Li-Chun et al. 2006), i.e., the short average distance in the graph between any two nodes. Similarly, the Internet is what we call a scale-free topology (Barabási et al. 2000; Zhang et al. 2011). Such networks have a few nodes with very many connections, called “hubs”, and very many nodes with only a few connections. In social networks, we would call the hubs “influencers”, while nodes with only a few connections are “followers”. For the question of where information comes from, a small-world graph topology means that an information can quickly spread and reach a wide audience taking only a few steps forward from the originator. From the consumer’s perspective, if the topology is scale-free (as the Internet appears to be), then it can most likely be traced back to one of the few hubs to be the originator. We find instances of this effect in many cases of news on the web, since newspapers often buy their information from larger news companies, so that articles, even if they appear in different media, may nonetheless originate from the same news agency. Tracing back the source of information is thus a crucial matter for judging the quality of information, as it can only be as good as the original source, and most likely, there are not too many independent such sources (and every forward can—even unintentionally—modify, degenerate or otherwise blur the information).

## Artificial intelligence (AI) as a remedy?

With all this known, it is tempting to ask if artificial intelligence could help us distinguish right from wrong information, but the challenge remains tricky. Certainly, a machine has—unlike a human—no self-interest or unethical intentions, but since AI draws its power from learning, it can be *trained* to serve unethical needs of a human designer. The point is that AI can only be as good as the data that has “shaped” it, e.g., recognizing or reproducing patterns that were in the training data. Consequently, AI will only be good in recognizing fake news if it has been trained on good set of reputable vs. fake cases. Likewise, it will only produce high quality and objective articles, if its training author provided such articles beforehand. The bottom line is that AI can at best be a supporting technology, not a substitute for human intelligence. The recently coined term “robot journalism” (Dörr 2016) uses the power of AI to machine-generate news articles. Can a human intelligence recognize this, say, if the AI were to publish biased (and hence in a way fake) news? Essentially yes: humans can be critical, reflect and challenge their own opinion and that of others. AI cannot do the same trick as good as a human, and as long as no AI has ever passed Turing’s famous test (which as of today has not happened), humans can recognize a machine talking to them with a chance of more than 50% in general.

So, is AI at all more a danger than useful to recognize fake news? The answer is clearly no, since the power of AI to recognize even the smallest inconsistencies in possibly huge lots of facts, or the ability to classify images based on features that are invisible for the human eye make it invaluable weapons for the recognition of fake news. “Stylometry” applies pattern recognition to linguistic data to assign authorship of an unknown text to a specific person (e.g., Ma et al. 2009; Holmes and Kardos 2003). Statistical methods like the Benford test (Durtschi et al. 2004) can recognize forgeries in numbers (in a weak form but enough to raise awareness to take a second look). The real power, as always, lies in a clever combination of such techniques.

## Final remarks

There is neither a silver bullet nor a one-size-fits-all algorithm to recognize fake news. Essentially it boils down to a battle of wits between those generating false facts, and those who shall buy it. The position made here is to raise awareness that the best practice to protect against fake information is to follow a scientific approach that, perhaps not surprisingly, also kids adopt by nature: question everything and always ask “who said that?”. Try to use more than one source of information, say, not only use Google, but use a *meta*-*searcher* instead, which can query Google but also independent other sources. Likewise, do not buy a picture “as is”, but rather check if it appears elsewhere too, perhaps in a different context. Numbers can appear more convincing that qualitative facts, but even a perfectly ethical and careful intention behind a statistic is no protection against human error when the numbers are computed. Statistics should always open the underlying data to let others judge and reproduce the results; an issue called the “scientific reproducibility crisis” (Stoddart 2016) today. The same applies for news vs. fake news: can the claimed facts be verified from independent sources, i.e., not only by getting many copies but getting the same data using a different origin? The question has no ultimate answer, but awareness is already a key step towards tackling the risk of fake news.
